# Changes in cardiovascular health score and atherosclerosis progression in middle-aged and older persons in China: a cohort study

**DOI:** 10.1136/bmjopen-2014-007547

**Published:** 2015-08-26

**Authors:** Jingsheng Gao, Minghui Bao, Yan Liu, Jihong Shi, Zhe Huang, Aijun Xing, Yang Wang, Shasha An, Jun Cai, Shouling Wu, Xinchun Yang

**Affiliations:** 1 Department of Cardiology, Kailuan Hospital, Hebei United University, Tangshan, China; 2 Department of Cardiology, Chaoyang Hospital, Capital Medical University, Beijing, China

**Keywords:** CARDIOLOGY

## Abstract

**Objectives:**

The American Heart Association (AHA) proposed a definition of 4 cardiovascular health behaviours and 3 health factors. On the basis of the 7 metrics, the cardiovascular health score (CHS) was used to estimate individual-level changes in cardiovascular health status. The aim of this study was to investigate whether changes in CHS (⊿CHS) at different time-points are associated with atherosclerosis progression in middle-aged and older persons.

**Design:**

Prospective cohort study in China.

**Settings:**

We defined 8 groups (≤−4, −3, −2, −1, 0, 1, 2 and ≥3) according to ⊿CHS. The impact of ⊿CHS on the change of brachial–ankle pulse wave velocity (⊿baPWV) and atherosclerosis progression was analysed.

**Participants:**

A total of 3951 individuals met the inclusion criteria (≥40 years old; no history of stroke, transient ischaemic attack or myocardial infarction) and had complete information.

**Results:**

⊿baPWV decreased gradually (126.46±355.91, 78.4±343.81, 69.6±316.27, 49.59±287.57, 57.07±261.17, 40.45±264.27, 37.45±283.26 and 21.66±264.17 cm/s, respectively) with increasing ⊿CHS (p for trend<0.05). Multivariate linear regression analysis suggested a negative relationship between these 2 variables, which persisted after adjustment for other risk factors. Each increase in CHS was associated with a reduced baPWV for 15.22 cm/s (B value −15.22, p<0.001).

**Conclusions:**

⊿CHS were negatively related to ⊿baPWV, which proved to be an independent predictor of the progression of atherosclerosis in middle-aged and older persons.

**Trial registration number:**

Kailuan study (ChiCTR-TNC-11001489).

**Strengths and limitations of this study**

- Measurements taken twice to estimate individual-level changes in cardiovascular health status.
- The present study explored the changes in cardiovascular health status, as well as investigated atherosclerosis progression which was evaluated by brachial–ankle pulse wave velocity.
- We did not adhere perfectly to all of the American Heart Association (AHA) 2020 health metrics for practical reasons.
- The duration of follow-up was 2 years, which was not long enough to fully track the progression of atherosclerosis.
- The results of the study are based on middle-aged and older persons, but whether it can be generalised to the whole population warrants further validation.

## Introduction

Cardiovascular disease (CVD) is the leading cause of morbidity and mortality worldwide.^1^ ^2^ Several risk factors, such as cigarette smoking, excessive alcohol consumption, imbalanced diet, obesity and physical inactivity, can promote the development of CVD and premature mortality.^3^ Avoiding these risk factors and adopting healthy lifestyle changes might result in a lower CVD incidence.^4^

In 2010, the American Heart Association (AHA) proposed its first definition of cardiovascular health behaviours and health factors.^2^ This definition consists of seven metrics—four health behaviours (smoking, diet, physical activity and body mass index (BMI)) and three health factors (plasma glucose, cholesterol and blood pressure (BP))—that are used to categorise individuals into ‘poor’, ‘intermediate’ and ‘ideal’ groups. To estimate individual-level changes in cardiovascular health behaviours and health factors, Huffman^5^ established the AHA cardiovascular health score (CHS), which includes all seven cardiovascular health behaviours and health factors (each factor scored as—poor, 0 point; intermediate, 1 point; or ideal, 2 points—total scale: 0–14 points). Several studies have since detected a protective effect of ideal cardiovascular health behaviours and health factors on the incidence of cardiovascular and cerebrovascular diseases. The risk of cardiovascular and cerebrovascular diseases, all-cause mortality and cardiovascular mortality dramatically declines with increasing values for cardiovascular health metrics.^3^ ^6^ Moreover, cardiovascular health status conversion from ‘poor’ or ‘intermediate’ to ‘ideal’ might also benefit the cardiovascular and cerebrovascular systems by reducing the odds of detectable coronary artery calcification and lowering the intima–media thickness.^7^ Unfortunately, both the prevalence of ideal cardiovascular health status and the improvement of CHS are far from satisfactory.^2^ ^8^

Atherosclerosis is a chief factor contributing to the occurrence and development of CVD. Arterial stiffness is a predictor of atherosclerosis and CVD,^9^ which can be evaluated by pulse wave velocity (PWV). Among multiple methods used to measure PWV,^10^ brachial–ankle PWV (baPWV) can be measured much more conveniently and non-invasively^11^ and has the potential to be widely used in large-scale clinical studies. Positive relationships between baPWV and CVD have been detected in several studies.^12–15^

Taking into consideration these findings, the protective effect of CHS on the incidence of CVD might be mediated by its favourable effects on baPWV. To the best of our knowledge, however, the relationship between CHS changes and baPWV changes has not been reported. Therefore, on the basis of the population of the Chinese Kailuan study (ChiCTR-TNC-11001489), we adopted baPWV as an index to investigate whether changes in CHS (⊿CHS) can affect the progression of atherosclerosis in middle-aged and older persons.

## Materials and methods

### Study participants

Eleven hospitals in China participated in the physical examination. A total of four physical examinations were performed during 2006–2007, 2008–2009, 2010–2011 and 2012–2013, respectively. The measurement of baPWV was added into the last two examinations. The examination was performed on both in-service and retired workers between June 2006 and October 2007. A total of 101 510 workers participated in the physical examination (81 110 men and 20 400 women).

#### Inclusion and exclusion criteria

Participants were included if they were ≥40 years old and provided informed consent for the present study. Participants were excluded if they had history of stroke (except for lacunars infarction), had history of transient ischaemic attack, had history of myocardial infarction, had incomplete information (cardiovascular health metrics, baPWV), had extreme values of baPWV at the third or the fourth examination or died during follow-up.

### Data collection

#### Epidemiological questionnaire

The questionnaire was completed by trained researchers or doctors on the day of the health examination. The questionnaire items consisted of demographic information, occupation situation, lifestyle (eg, cigarette smoking, exercise and diet), disease history and family history, and physical examination profiles (eg, BP, height, weight, waist circumference, etc.). Smoking was defined as ≥1 cigarette/day, continuous smoking ≥1 year, or giving up smoking ≤1 year.

#### Anthropometric and biochemical measurements

Standard protocols were used for all the measurements as described earlier by our group.^16^ Anthropometric measurements included the measurements of height, weight, waist circumference, hip circumference, BMI, and BP. Biochemical measurements: fasting blood glucose (FBG), triglyceride (TG), total cholesterol (TC), high-density lipoprotein cholesterol (HDL-C), low density lipoprotein cholesterol (LDL-C), etc, (see online supplementary method 1 for details).

#### baPWV measurement

Measurements were repeated twice for each people and the second data was regarded as the final record. We analysed the larger value of the left and right side. The changes of baPWV (⊿baPWV) were calculated as the baPWV value in examination of 2012–2013 minus the value in 2010–2011. Individuals were divided into 2 groups according to ⊿baPWV. Participants with ⊿baPWV≤0 were defined as non-increasing group, and participants with ⊿baPWV>0 were defined as increasing group (see online supplementary method 2 for details).

#### Cardiovascular health metrics

According to the cardiovascular health behaviours and health factors proposed by AHA,^2^ and the scoring system (the AHA CHS) raised by Huffman,^5^ the seven cardiovascular health behaviours and health factors were divided into three levels (poor=0 point; intermediate=1 point; and ideal=2 points—total scale: 0–14 points). Owing to lacking of detailed diet data in the original scale, and taken into account the great influence of salt intake on CVD among Chinese population, we adopted salt intake as a surrogate of diet. The definition of exercise is slightly different with which proposed by AHA (the ideal amount of exercise proposed by AHA is more than 5 times per week and more than 30 min each time) (see online supplementary method 3 for details).

#### Grouping method

We adopted the scoring system named CHS proposed by Huffman. Assignment of cardiovascular health metrics: poor, 0 point; intermediate, 1 point; ideal, 2 points—total scale: 0–14 points. The ⊿CHS: the CHS in examination of 2012–2013 minus the CHS in 2010–2011. We divided population into eight groups (≤−4, −3, −2, −1, 0, 1, 2 and ≥3) and classified them into three groups: decreasing group (⊿CHS<0), invariant group (⊿CHS=0), and increasing group (⊿CHS>0).

#### Data management and statistical method

EpiData software was used to establish the data base. SPSS 13.0 statistical software was used for statistical analysis. Normally distributed data was recorded as (mean±SD). Variance analysis was used when more than two groups were compared. The mean was compared by the method of Least Significant Difference (LSD) (homogeneous variance) or Welch (heterogeneous variance). Categorical variables were described as percentages and compared using χ^2^. Multivariate linear regression analysis was used to investigate the relationship between ⊿CHS and ⊿baPWV. The p<0.05 (bilateral) was regarded as statistically significant.

#### Missing values replacement

We adopted data of the first, second and third physical examination as replacement of the missing values of the third and fourth examination. The extreme value of baPWV refers to values beyond the 99 centile. The extreme value of the third examination is 2829 cm/s (the left side) and 2707 cm/s (the right side). The extreme value of the fourth examination is 3734 cm/s (the left side) and 3955 cm/s (the right side) (see online supplementary method 4 for details). In order to reduce the bias and improve the authenticity of our results, we excluded the extreme values of baPWV for the consideration that these extreme values might be due to the measurement errors.

## Results

**Figure 1:**
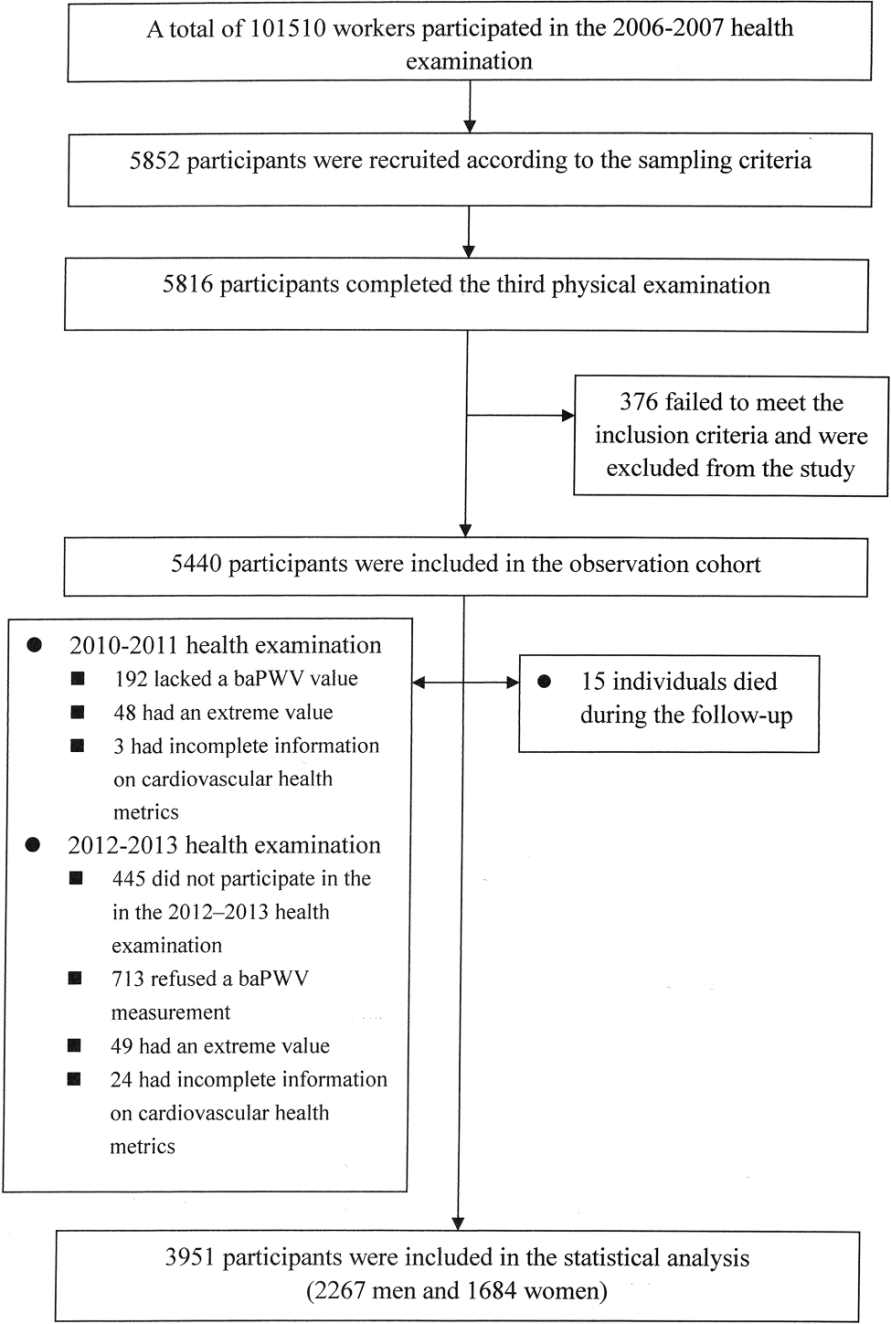
Flow chart of this study (baPWV, brachial–ankle pulse wave velocity).

From 2010 to 2011, a sample of 5852 participants older than 40 years was randomly selected from 101 510 workers. In total, 5816 participants eventually completed the third physical examination. Among them, 376 participants did not meet the following inclusion criteria: no history of stroke, transient ischaemic attack or myocardial infarction. Of the remaining 5440 individuals, 445 did not participate in the fourth examination. About 243 had incomplete information (baPWV, cardiovascular health metrics) and extreme value of baPWV at the third examination, and 786 at the fourth examination. Fifteen individuals died during the follow-up. Finally, 3951 participants were included in the statistical analysis (2267 men and 1684 women). See figure 1 for detailed information of participants inclusions and exclusions. See online supplementary table S1 for detailed baseline characteristics of included and excluded participants.

### Baseline characteristics of different groups

Since measurement of baPWV began with the 2010–2011 physical examinations, we used data from 2010 to 2011 as the baseline for this study. After the 2-year follow-up, the percentages of groups with a decreased, unchanged and increased CHS values were 44.57%, 22.07%, and 33.36%, respectively. Baseline characteristics of groups with different ⊿CHS are shown in table 1. The average age was 53.66±10.97 years, and 57.38% were men. The systolic BP (SBP), diastolic BP (DBP), BMI, TC and FBG were in normal ranges. After 2 years of follow-up, the SBP, TC, FBG and baPWV had increased from baseline; however the DBP, BMI and CHS had decreased from baseline. With ⊿CHS converted from≤−4 to ≥3, all of the items showed an increasing trend (p for trend<0.001) while the baseline age reduced gradually. In groups with an increased CHS (⊿CHS=1, ⊿CHS=2 and ⊿CHS≥3), a more obvious increase of ⊿CHS resulted in a more significant reduction of ⊿SBP, ⊿DBP, ⊿BMI, ⊿TC and ⊿FBG.

**Table 1:** Baseline characteristics of groups with different ⊿CHS (n=3951)

	Total	≤−4	−3	−2	−1
n	3951	171	286	506	798
*Baseline (2010–2011)*					
Age, year	53.66±10.97	57.04±10.6	55.19±11.06	53.86±11.37	54.32±11.22
Male (%)	2267 (57.38)	84 (49.12)	140 (48.95)	265 (52.37)	426 (53.38)
SBP, mm Hg	129.73±19.27	124.73±19.09	126.72±20.28	127.23±19.57	128.52±20.51
DBP, mm Hg	82.73±10.89	77.97±10.12	80.84±10.79	80.67±11.05	81.3±10.74
BMI, kg/m^2^	24.96±3.22	24.69±3.02	24.92±3.05	24.57±3.22	24.86±3.18
TC, mg/dL	195.77±38.59	190.09±37.47	190.9±34.04	190.47±34.22	194.72±36.71
FBG, mg/dL	99.88±25.35	97±27.7	96.95±23.69	97.61±24.98	99.43±24.11
CHS	8.6±2.32	10.45±1.74	10.04±1.87	9.61±2.04	9.1±2.2
*Changes (2012–2013, 2010–2011)*					
⊿SBP, mm Hg	1.81±17.44	12.42±19.63	8.14±16.29	6.78±17.05	3.1±16.7
⊿DBP, mm Hg	−0.46±12.12	8.24±14	3.62±11.37	2.38±11.84	1.5±11.54
⊿BMI, kg/m^2^	−0.04±2.36	1.6±4.05	0.57±2.33	0.31±2.5	0.16±2.05
⊿TC, mg/dL	4.33±52.25	27.81±32.41	25.36±117.91	15.73±31.05	7.41±33.64
⊿FBG, mg/dL	3.2±22.1	14.38±26.13	13.1±27.77	8.23±21.23	4.86±21.29
⊿CHS	−0.28±1.88	−4.42±0.77	−3±0	−2±0	−1±0

	**0**	**1**	**2**	**≥3**	**p Value**

n	872	681	397	240	
*Baseline (2010–2011)*					
Age, years	53.42±10.93	52.7±10.58	52.61±10.61	52.1±10.57	<0.001
Male (%)	510 (58.49)	412 (60.5)	262 (65.99)	168 (70)	<0.001
SBP, mm Hg	130.71±19.41	131.09±18.6	132.19±16.83	134.69±15.97	<0.001
DBP, mm Hg	83.23±10.88	84.12±10.7	85.61±10.05	87.03±10.28	<0.001
BMI, kg/m^2^	24.93±3.31	25.07±3.24	25.36±3.21	25.55±3.21	0.001
TC, mg/dL	195.29±40.31	196.53±39.1	202.56±42.12	208.61±41.2	<0.001
FBG, mg/dL	98.03±21.92	101.65±26.31	103.58±27.86	107.34±31.85	<0.001
CHS	8.44±2.11	7.98±2.11	7.25±2.07	6.27±2.06	<0.001
*Changes (2012–2013, 2010–2011)*					
⊿SBP, mm Hg	1.09±16.6	−1.57±16.47	−3.14±16.87	−7.72±17.04	<0.001
⊿DBP, mm Hg	−0.69±11.44	−3.34±10.99	−4.59±11.44	−8.17±11.91	<0.001
⊿BMI, kg/m^2^	−0.18±1.82	−0.39±2.01	−0.67±2.08	−0.86±3.37	<0.001
⊿TC, mg/dL	0.85±30.18	−0.97±66.6	−10.25±38.6	−20.02±35.64	<0.001
⊿FBG, mg/dL	3.04±18.73	−0.5±17.93	−4.62±17.82	−8.65±30.58	<0.001
⊿CHS	0±0	1±0	2±0	3.5±0.81	<0.001

changes (⊿), physical examination metrics of 2012–2013 minus metrics for 2010–2011; baPWV, brachial–ankle pulse wave velocity; BMI, body mass index; CHS, cardiovascular health score; DBP, diastolic blood pressure; FBG, fasting blood glucose; SBP, systolic blood pressure; TC, total cholesterol.

### baPWV of groups with different ⊿CHS

The baseline baPWV, follow-up baPWV, and ⊿baPWV of groups with different ⊿CHS are shown in table 2. The groups did not differ significantly from one another (p=0.507). With ⊿CHS converted from ≤−4 to ≥3 (⊿CHS≤−4, =−3, =−2, =−1, =0, =1, =2, and ≥3), ⊿baPWV reduced gradually (126.46±355.91, 78.4±343.81, 69.6±316.27, 49.59±287.57, 57.07±261.17, 40.45±264.27, 37.45±283.26 and 21.66±264.17 cm/s, respectively; p for trend<0.05). A more obvious increase in ⊿CHS was associated with a steeper reduction in ⊿baPWV (see table 1 for details). In addition, when ⊿CHS≥4, the baPWV did not increase further but rather decreased gradually (−29.90±215.92 cm/s) (see online supplementary table S2 for details). Although no statistical significance was achieved, we still detected a trend of an inverse relationship between ⊿CHS and ⊿baPWV.

**Table 2:** baPWV of groups with different ⊿CHS

	Total	≤−4	−3	−2	−1
N	3951	171	286	506	798
Baseline baPWV, cm/s	1535.04±336.88	1562.47±314.23	1567.21±368.95	1538.97±336.57	1535.63±352.98
Follow-up baPWV, cm/s	1589.77±403.25	1688.92±436.74	1645.61±458.72	1608.58±436.8	1585.21±404.86
⊿baPWV, cm/s	54.72±288.47	126.46±355.91	78.4±343.81	69.6±316.27	49.59±287.57
⊿baPWV groups					
Non-increasing group, n (%)	1659 (41.99)	67 (39.18)	117 (40.91)	206 (40.71)	341 (42.73)
Increasing group, n (%)	2292 (58.01)	104 (60.82)	169 (59.09)	300 (59.29)	457 (57.27)

	0	1	2	≥3	p Value
n	872	681	397	240	
Baseline baPWV, cm/s	1529.53±335.94	1528.66±336.06	1533.7±322.67	1507.29±283.35	0.507
Follow-up baPWV, cm/s	1586.6±395.12	1569.11±385.76	1571.14±369.94	1528.95±339.08	0.001
⊿baPWV, cm/s	57.07±261.17	40.45±264.27	37.45±283.26	21.66±264.17	0.005
⊿baPWV groups					0.518
Decreasing group, n (%)	364 (41.74)	286 (42)	168 (42.32)	110 (45.83)	
Increasing group, n (%)	508 (58.26)	395 (58)	229 (57.68)	130 (54.17)	

⊿baPWV, baPWV of the physical examination in 2012–2013 minus baPWV of 2010–2011; baseline baPWV, baPWV of physical examination in 2010–2011; follow-up baPWV, baPWV of the physical examination in 2012–2013; increasing group, ⊿baPWV>0; non-increasing group,

⊿baPWV≤0. baPWV, brachial–ankle pulse wave velocity; CHS, cardiovascular health score; ⊿CHS, CHS of the physical examination in 2012–2013 minus CHS in 2010–2011.

### Linear regression analysis between ⊿CHS and ⊿baPWV

The linear regression analysis was performed with ⊿baPWV as a dependent variable and ⊿CHS as the independent variable. Mode 1: This was a single-factor analysis model. Mode 2: On the basis of Mode 1, we further adjusted for age and gender. Mode 3: On the basis of Mode 2, we further adjusted for baseline CHS and baseline baPWV. The results of mode 3 suggested that age were positively associated with ⊿baPWV. Female gender, baseline CHS, baseline baPWV, and ⊿CHS were negatively associated with ⊿baPWV. With adjustments for age, gender, baseline CHS, and baseline baPWV, ⊿baPWV decreased with increasing ⊿CHS. Each increase in ⊿CHS was associated with a 15.22 cm/s decrease in ⊿baPWV (B value −15.22, p<0.001; table 3). The relationships between each individual cardiovascular health component and ⊿baPWV were also analysed by using linear regression model (see online supplementary table S3 for details).

**Table 3:** Linear regression analysis between ⊿CHS and ⊿baPWV

	B value	95% CI	β	p Value
Mode 1				
⊿CHS	−10.06	−14.84 to −5.27	−0.07	0.000
Mode 2				
⊿CHS	−10.56	−15.41 to −5.71	−0.07	0.000
Baseline age	0.62	−0.22 to 1.46	0.02	0.150
Female	−27.37	−46.03 to −8.72	−0.05	0.000
Mode 3				
⊿CHS	−15.22	−20.43 to −10.02	−0.10	0.000
Baseline age	6.76	5.71 to 7.81	0.26	0.000
Female	−28.28	−47.73 to −8.84	−0.05	0.004
Baseline CHS	−10.98	−15.68 to −6.29	−0.09	0.000
Baseline baPWV	−0.32	−0.36 to −0.29	−0.38	0.000

⊿baPWV, baPWV of the fourth physical examination minus baPWV of the third physical examination; ⊿CHS, CHS of the fourth physical examination minus CHS of the third physical examination; mode 1, a single-factor analysis model; mode 2, adjusted for age and gender on the basis of mode 1; mode 3, adjusted for baseline CHS and baseline baPWV on the basis of mode 2.

baPWV, brachial–ankle pulse wave velocity; CHS, cardiovascular health score.

### Sensitivity analysis

To eliminate the influence of missing value replacement, we excluded participants with missing values and performed a statistical analysis of data without replacement. The results remain unchanged. The new analysis showed a result similar to that of the original analysis and indicated an obvious negative relationship between ⊿CHS and ⊿baPWV. With adjustments for age, gender, baseline CHS and baseline baPWV, each increase in ⊿CHS was associated with an 11.32 cm/s decrease in ⊿baPWV (p<0.001; table 4).

**Table 4:** Sensitivity analysis

	B value	95% CI	β	p Value
Mode 3				
⊿CHS	−11.32	−17.25 to −5.40	−0.08	0.000
Baseline age	6.83	5.48 to 8.18	0.23	0.000
Female	−43.63	−66.40 to −20.87	−0.08	0.004
Baseline CHS	−9.15	−14.82 to −3.48	−0.08	0.002
Baseline baPWV	−0.31	−0.35 to −0.27	−0.34	0.000

⊿baPWV, baPWV of the fourth physical examination minus baPWV of the third physical examination; ⊿CHS, CHS of the fourth physical examination minus CHS of the third physical examination; mode, adjusted for multiple factors.

baPWV, brachial–ankle pulse wave velocity; CHS, cardiovascular health score.

## Discussion

Since the AHA proposed seven metrics of cardiovascular health behaviours and health factors, several studies have suggested that ideal cardiovascular health metrics plays an important role in protection of the cardiovascular and cerebrovascular systems.^3^ ^6^ With an increasing value of cardiovascular health metrics, high-sensitivity C reactive protein,^17^ carotid artery intima–media thickness^18^ ^19^ and the incidence of cardiovascular and cerebrovascular events^3^ ^6^ decrease gradually. In addition, improvement in cardiovascular health status can reduce the risk of subclinical atherosclerosis and cardiovascular events.^7^ ^20^ Arterial stiffness is a predictor of atherosclerosis and CVD,^9^ which can be measured by PWV.^10^ Carotid-femoral pulse wave velocity (cfPWV) is the gold standard for measuring arterial stiffness and is reported to predict future mortality^21^ and morbidity^9^ from CVD, but its measurement is fairly complicated and time-consuming. However, baPWV measures have an excellent correlation with cfPWV and can reflect the elasticity of the large and the medium arterial systems; furthermore, baPWV measurement is much more convenient and requires only a short period of time.^11^ The relationships between baPWV and CVD mortality in the older population, total mortality in the general population,^12^ CVD incidence^14^ and CVD risk^15^ have been reported in several studies. It is most likely that the protective effect of cardiovascular health status improvement on the incidence of CVD might be mediated by its favourable effects on baPWV.

The influencing factors of baPWV are rather complicated. Numbers of metabolic syndrome components,^22^ body size phenotypes,^23^ ageing and male gender,^24^ aerobic exercise and smoking^25^ as well as BP control^26^ were reported to impact on baPWV. Therefore, evaluating the effect of each factor is difficult to achieve. For this reason, a comprehensive estimate system is needed to investigate the relationship between baPWV and multiple influencing factors. The AHA CHS, based on four health behaviours and three health factors, covers a variety of influences. Thus, analysing the relationship between ⊿CHS and ⊿baPWV might be a useful tool, but the relationship between them has rarely been reported. As far as we know, our study is the first large-scale investigation aimed at exploring this association. The outcomes of our study indicated that ⊿baPWV decreased gradually with increasing ⊿CHS. In addition, a more obvious CHS decrease was associated with a more significant baPWV increase, and conversely, a more obvious CHS increase resulted in attenuation of the baPWV increase. Indeed, at⊿CHS≥4, baPWV stopped increasing and began a gradual decrease. These results suggest that an improved CHS can reduce the progression of atherosclerosis, as measured by baPWV. The negative relationship between ⊿CHS and ⊿baPWV remains unchanged after adjusting for age, gender, baseline CHS and baseline baPWV. Each increase in ⊿CHS was associated with a ⊿baPWV decrease of 15.22 cm/s. Previous studies have shown that atherosclerosis is a reversible process. Diet, regular exercise and lipid-lowering treatment might reverse the progression of atherosclerosis.^27^ ^28^ Our present findings confirmed this as well: individuals with increased CHS experienced a reduced atherosclerosis progression. Moreover, the progression was reversed among people with ⊿CHS≥4.

Previous work from Aatola *et al*^29^ estimated changes in ideal cardiovascular health status among children and youth. The results showed that a change in ideal cardiovascular health status, from childhood or young adulthood, was an independent predictor of adult PWV; however, whether the beneficial effect could also be found in middle-aged and older people was unknown. The current results suggest that an improvement in cardiovascular health status plays an important role for middle-aged and older people, with a favourable effect that appears in a short period of time. Low prevalence and poor improvement of ideal cardiovascular health was reported in several studies. Only 5% of the population meets the criteria for ideal cardiovascular health in the USA.^2^ The percentage of individuals with an increased CHS in the CARDIA study was 25.3%.^7^ A similar trend was also found in China by Wu *et al.*^8^ Only 0.6% of male participants and 2.6% of female participants (n=1 012 418) met the ‘ideal’ for all seven health components. The reason for a higher improvement ratio (33.36%) in our study might be the regular health education and physical examination among workers in the Kailuan Group Corporation. If this practice and experience were generalised to the entire Chinese population, a more significant benefit to the cardiovascular and cerebrovascular system might be achieved.

This study has some limitations. First, we did not completely adhere to the health indicators proposed by AHA. Since there were no specific data about diet, we adopted salt intake as a proxy, which could have led to an underestimate of the influence of diet on ⊿baPWV; however, evidence has shown that salt intake among Chinese people is much higher than in other countries.^30^ Thus, the amount of salt intake is likely to be of greater significance than other indicators of diet in the Chinese population. Second, the duration of follow-up was not long enough to fully track the progression of atherosclerosis. Nevertheless, a favourable effect of an improved CHS emerged even during the relatively short follow-up. Third, the results of our study are based on middle-aged and older people, but whether it can be generalised to the whole population warrants further validation.

In this prospective cohort study, we offer the first evidence of an inverse relationship between CHS improvement and baPWV. Each increase in CHS was associated with a reduced baPWV of 15.22 cm/s, which proved to be an independent predictor of atherosclerosis progression. These findings might partly explain the negative association between CHS and CVD incidence. Therefore, improving CHS and maintaining a healthy lifestyle can attenuate or even reverse the progression of atherosclerosis, which might partly explain the mechanism of an inverse relationship between CHS and incidence of CVD.
